# Plant-Derived Fermented Products: An Interesting Concept for Human Health

**DOI:** 10.1016/j.cdnut.2024.102162

**Published:** 2024-04-16

**Authors:** Danial Khayatan, Kiana Nouri, Saeideh Momtaz, Basil D Roufogalis, Mona Alidadi, Tannaz Jamialahmadi, Amir Hossein Abdolghaffari, Amirhossein Sahebkar

**Affiliations:** 1 Department of Toxicology and Pharmacology, Faculty of Pharmacy, Tehran Medical Sciences, Islamic Azad University, Tehran, Iran; 2 Medicinal Plants Research Center, Institute of Medicinal Plants, ACECR, Karaj, Iran; 3 Discipline of Pharmacology, School of Medical Sciences, University of Sydney, Sydney, New South Wales, Australia; 4 Department of Nutrition, Faculty of Medicine, Mashhad University of Medical Sciences, Mashhad, Iran; 5 Pharmaceutical Research Center, Pharmaceutical Technology Institute, Mashhad University of Medical Sciences, Mashhad, Iran; 6 Medical Toxicology Research Center, Mashhad University of Medical Sciences, Mashhad, Iran; 7 Center for Global Health Research, Saveetha Medical College and Hospitals, Saveetha Institute of Medical and Technical Sciences, Saveetha University, Chennai, India; 8 Biotechnology Research Center, Pharmaceutical Technology Institute, Mashhad University of Medical Sciences, Mashhad, Iran; 9 Applied Biomedical Research Center, Mashhad University of Medical Sciences, Mashhad, Iran

**Keywords:** plant-derived fermented products, fermentation, microorganisms, clinical studies

## Abstract

The health benefits of fermenting plant-derived products remain an underexplored domain. Plants and other natural products serve as medicinal agents when consumed as part of our diets, and the role of microorganisms in fermentation garners significant scientific interest. The present narrative review investigates the effects of fermentation of substances such as plants, algae, and fungi on their therapeutic and related purposes. Among the microorganisms used in fermentation, lactic acid bacteria are often linked to fermented products, particularly dairy and animal-based ones, and take center stage. These microorganisms are adept at synthesizing vitamins, active peptides, minerals, proteinases, and enzymes. Plant-derived fermented products are a significant source of active peptides, phytochemicals, flavonoids, and bioactive molecules with a profound impact on human health. They exhibit anti-inflammatory, anticarcinogenic, antiatherosclerotic, antidiabetic, antimicrobial, and antioxidant properties, the effects being substantiated by experimental studies. Clinical investigations underscore their effectiveness in managing diverse health conditions. Various studies highlight a synergy between microorganisms and plant-based materials, with fermentation as an innovative method for daily food preparation or a treatment option for specific ailments. These promising findings highlight the need for continued scientific inquiry into the impact of fermentation-derived products in clinical settings. Clinical observations to date have offered valuable insights into health improvement for various disorders. This current narrative review explores the impact of natural and plant-originated fermented products on health and well-being.

## Introduction

The prevention of neurodegenerative and age-related illnesses, such as liver disease, cancer, stroke, diabetes, neurodegeneration, and cardiovascular diseases (CVDs), has led to a substantial increase in new medical research. These conditions can have a significant impact on the quality of life (QOL) [1]. Fermented foods (FFs), fermented plant beverages (FPBs), and their extracts are typically defined as products resulting from interactions with microbial organisms, including bacteria, yeasts, and mycelial fungi, as well as their associated enzymes, in a process known as fermentation [2]. The fermentation process encompasses techniques such as salting, smoking, and drying, making it one of the oldest methods for preserving food and beverages to enhance their nutritional value. Fermentation is a technique that stimulates and regulates the growth and metabolic functions of microorganisms for the transformation and preservation of raw materials [3]. There are 2 primary types of fermentation. The first is natural fermentation, in which microorganisms are already part of the natural microflora found in the food or plant material. In this case, all that is required is creating a suitable environment for these microorganisms to thrive while inhibiting the growth of competing microflora. The second type is controlled fermentation, which involves deliberate inoculation with specific starter cultures containing a higher concentration of required fermentation microorganisms. Controlled fermentation becomes necessary when conditions are not ideal for the growth of fermentation microorganisms or when the raw materials have been pasteurized. These starter cultures include the natural fermentation agents present in the food's microflora but at significantly higher concentrations, ensuring their dominance over competing, spoilage-causing microorganisms [4]. The fermentation process involves several common microorganisms, including lactic acid bacteria (LABs), propionibacterium, acetobacter, yeast, molds, and bacillus species. These microorganisms play a crucial role in producing various compounds such as lactic acid, acetic acid, propionic acids, alcohol, ammonia, and fatty acids, which contribute to the unique characteristics of fermented products [5,6]. Historically, fermented foods and beverages were primarily used to extend the shelf life of plant-based and animal-derived raw materials. Today, besides enhancing shelf life, this bioprocess technology is also employed to improve sensory qualities, nutritional properties, and overall food safety [7,8]. Fermented foods have recently gained popularity, ranking among the top 10 food trends. Food companies are responding by either developing new FF based on traditional recipes or commercializing traditional FF, such as kombucha and kefir. There is a growing market for soy-based or cereal-based probiotic products, driven by factors such as dairy allergies, gluten or lactose intolerances, and lifestyle choices such as veganism [9,10]. The appeal of fermented products has captured the attention of scientists, leading to expanded research efforts in this field. Some fermented products, recognized as FF, are valued for their health-supportive properties, including anti-inflammatory, antihypertensive, antimicrobial, antioxidant, and hypolipidemic functions. Consequently, plant-derived fermented products have garnered significant interest from both the scientific community and health-conscious consumers. This growing interest has fueled numerous investigations focusing on plant-derived fermented products from various health perspectives [11].

The notable surge in the popularity of fermented plant-based food products has led to these items being commonly purchased and consumed by a diverse spectrum of individuals in various demographic groups. Health-conscious consumers, including vegetarians, vegans, and those with dietary preferences or restrictions, often seek out these products for their nutritional benefits and ethical considerations. In addition, individuals looking to enhance their gut health and overall well-being are drawn to the potential health advantages of fermented plant-based foods. People with lactose intolerance or dairy allergies turn to these dairy-free alternatives, whereas vegans find them aligned with their ethical and environmental values. Additionally, regions with cultural traditions of fermented plant-based foods continue to contribute to their popularity. The appeal extends to those with gluten sensitivity and individuals committed to reducing their environmental footprint. In essence, the consumption of fermented plant-based foods spans a wide array of demographic groups, reflecting the diversity of dietary preferences, health concerns, and ethical considerations among consumers and thereby capturing the attention of a broad audience [12].

In this context, fermented plant extracts (FPEs) emerge as noteworthy plant functional foods, many originating in Japan. These extracts are rich sources of nutrients and active substances, such as antioxidants, vitamins, minerals, polyphenols, proteins, fibers, and probiotics [13,14]. FPEs exert a multifaceted impact on various health conditions, influencing the overall well-being of the human body through various signaling pathways. Research has unveiled several health benefits associated with FPEs in animals, encompassing cardioprotective, antifungal, antioxidative, anti-inflammatory, lipid-lowering, and antidiabetic properties. Consequently, industrial companies have turned their attention to the production of formulated beverages or juices as plant-derived fermented products, with a specific focus on extending shelf life and stabilizing formulations.

This narrative review investigates the latest data regarding the advantages of plant-derived fermented products used in various health conditions, offering insights into their effects on well-being. It also engages in a comprehensive discussion of the detailed mechanisms of action, with a specific focus on clinical trials. For transparency and methodological rigor in our study, our narrative review aimed to encompass a wide spectrum of research findings related to plant and natural product-derived fermented products and their health implications. To ensure the inclusivity of our research, we executed a thorough and unbiased literature search, leveraging key academic databases such as PubMed, Scopus, and Web of Science. Employing a comprehensive set of specific keywords related to fermented plant-based items, our strategy was carefully designed to minimize risk of selection bias and to incorporate a diverse range of studies into our analysis. Our search strategy employed a comprehensive set of specific keywords related to fermented plant-based items. “*Laminaria japonica*,” Plant-derived fermented products, fermentation, microorganisms, “*Saccharina japonica*,” “Turmeric,” “*Curcuma longa*,” *Camellia sinensis*, Tea, *Chamaecyparis obtuse*, *Carica papaya* L, Fermented wheat germ extract, Glycine max, rice koji, *Panax ginseng*, *Cyclopia intermedia*, and Bilberry (*Vaccinium myrtillus* L.) were carefully selected to encompass a wide range of relevant literature. Clear inclusion and exclusion criteria were established to guide the selection of studies for review. These criteria ensured that only relevant studies meeting predetermined quality standards were included.

## Fungal and Alga-Derived Fermented Products

### **Laminaria japonica**

*Laminaria japonica*, also called “Sea tangle,” is found in the coastal regions of Korea. These edible brown marine plants are composed of minerals, vitamins, lipids, protein, and carbohydrates, although their nutrient content varies depending on geographical position, temperature, species, and season. Studies revealed diverse biological properties of sea tangles, including antibacterial, antimutagenic, and antioxidant activities [15]. In a 6-wk randomized controlled trial (RCT), Reid et al. [16] assessed the effect of 1.5 g/d fermented sea tangle (FST) on 40 senior subjects. At the end of the study, neuropsychological test scores, including the Korean-mini-mental status examination (K-MMSE), Raven test, numerical memory test, and iconic memory test, were significantly improved compared with the placebo group. Moreover, the FST treatment increased the serum brain-derived neurotrophic factor (BDNF) and insulin-like growth factor 1 (IGF-1) concentrations, boosted the antioxidant activity of superoxide dismutase (SOD), glutathione peroxidase (GPx), and glutathione-disulfide reductase (GSR), whereas thiobarbituric acid reactive substances (TBARS) (biomarkers of lipid peroxidation), and 8-oxodG (oxidative DNA damage) were lowered. In this trial, 6-min walk test time, an indication of physical function, was significantly improved [1]. In another 2 RCTs, the effects of (1.5 g/d) FST on 48 healthy male volunteers with high concentrations of gamma-glutamyl transferase (γ-GT) were assessed during 4 wk of intervention [17,18]. In the first study, the FST-treated group demonstrated a marked reduction in serum concentrations of γ-GT and malondialdehyde (MDA) and a significant augmentation in SOD and catalase (CAT) activities compared with the placebo group, and FST increased the antioxidant defense system in a healthy population [17]. In the second study, the concentrations of γ-GT, aspartate aminotransferase (AST), and alanine transaminase (ALT) in the FST group were drastically lower than in the placebo group. Moreover, the concentrations of lactate dehydrogenase (LDH), blood urea nitrogen (BUN), 8-isoprostane, 8-hydroxy-2'-deoxyguanosine (8-OHdG), and protein carbonyl were decreased following the FST administration compared with the baseline. Lactobacillus casei (LTL1879) enhances the efficacy of oxidation and the intestinal microbial content of healthy and young volunteers. Also, LTL1879 demonstrated an effective function in decreasing MDA, increasing SOD, and reducing TNF-α and IL-10 [18]. Within plant-derived fermented products, the intriguing partnership between LTL1879 and Laminaria japonica, a type of brown seaweed, is a noteworthy example of how specific microbial strains can intricately shape the characteristics and potential health benefits of the final product. Lactobacillus casei LTL1879, a well-documented probiotic strain known for its health-promoting attributes, plays a pivotal role in the fermentation of Laminaria japonica. This strain, selected for its unique metabolic capabilities, actively participates in the transformation of the seaweed's compounds during the fermentation process. Laminaria japonica itself is rich in bioactive compounds, including polysaccharides and phenolic compounds, which are known for their potential health benefits. Through the action of LTL1879, these compounds undergo various bioconversions, leading to the production of metabolites such as bioactive peptides, organic acids, and potentially enhanced concentrations of beneficial polysaccharides. The interaction between the probiotic strain and the seaweed matrix can result in a synergistic effect, further enriching the fermented product with health-promoting molecules. The intricate metabolic pathways and interactions involved in this fermentation process offer a fascinating avenue for research and exploration, promising novel insights into the potential health benefits of Laminaria japonica-based fermented products [18].

### *Saccharina japonica*

*Saccharina japonica*, belonging to the Phaeophyceae (brown algae) family, has its origins mainly in Asian countries, particularly China, Japan, and Korea. It was important not only as readily accessible food but also as natural medicine for diseases. Certain polysaccharides found in brown algae, such as laminarans, alginic acids, and sulfated polysaccharides (fucoidans), have shown efficacy in cancers of the colon and breast [19]. Some studies have indicated that regular consumption of *Saccharina japonica* may help reduce risk of colon and breast cancers. This preventive effect could be attributed to its rich content of bioactive compounds, including various polysaccharides, polyphenols, and other antioxidants. These compounds are known for their potential to inhibit the growth and proliferation of cancer cells, scavenge free radicals, and modulate inflammatory processes, all of which contribute to cancer prevention. *Saccharina japonica* contains unique bioactive molecules, such as fucoidans, which have demonstrated antitumor properties. These substances may interfere with the growth of cancer cells, induce apoptosis (programmed cell death), and inhibit angiogenesis (the formation of new blood vessels that feed tumors). This suggests that *Saccharina japonica* may be explored as a complementary therapy in the treatment of colon and breast cancers. Some research suggests that *Saccharina japonica* may have immunomodulatory effects [20]. A well-functioning immune system plays a crucial role in detecting and eliminating cancerous cells. The seaweed’s components might enhance immune responses, aiding in the recognition and destruction of cancer cells in the colon and breast [21]. Chronic inflammation is associated with an increased risk of cancer. *Saccharina japonica* has demonstrated anti-inflammatory effects, potentially mitigating the chronic inflammation that can contribute to the development and progression of colon and breast cancers. It is important to note that although these findings are promising, more extensive research is needed to confirm the efficacy of Saccharina japonica in preventing and treating these cancers in humans. The mechanisms through which it exerts these effects also require further elucidation [22]. An RCT evaluated the efficacy of fermented S. japonica extract (FSJ) on working memory during working memory processing. Sixty-nine healthy volunteers were randomly assigned to consume 1 g/d of FSJ or placebo capsules for 4 wk. In the study, the FSJ group demonstrated statistically significant improvements in various aspects of cognitive function. Specifically, they exhibited a 15% increase in the percentage of correct answers during working memory processing and a 10% improvement in their ability to concentrate. Moreover, the FSJ group displayed significant alterations in left and right brain activity related to spatial perception, as assessed by the Raven test. These changes collectively indicate substantial enhancements in cognitive function. However, cognitive function assessment did not show marked differences between the groups, including the Korean Wechsler Adult Intelligence Scale, operation-word span task, and Raven’s test-based quantitative electroencephalogram (EEG) tests. The serum concentrations of amyloid-β and SOD tended to improve by 32% and 20%, respectively, for the FSJ group, and the improvement probably happened via regulation of the SOD antioxidant system [23].

## Plant-Derived Fermented Products

### *Curcuma longa (Zingiberaceae)*

Turmeric (often referred to as *Curcuma longa*) belongs to the Zingiberaceae family. Turmeric is a perennial herb with substantial antioxidant properties in comparison with standard antioxidants. The dried rhizomes and root powder of this member of the ginger family are utilized as a spice in various curries. Turmeric contains 3 significant compounds, namely demethoxycurcumin (DMC), bisdemethoxycurcumin (BDMC), and curcumin. Curcumin is known to have multiple biological and pharmacological actions [[24], [25], [26], [27], [28], [29], [30], [31], [32], [33], [34]], including potent anti-inflammatory and antioxidant effects [[35], [36], [37]]. In a randomized, double-blind, placebo-controlled trial conducted between November 2010 and April 2012, 60 subjects with mild-to-moderate elevated ALT concentrations (between 40 IU/L and 200 IU/L) were studied to investigate the hepatoprotective effects of fermented turmeric powder (FTP). The participants were divided into 2 groups, one receiving 3.0 g of FTP per day and the other receiving a placebo for 12 wk. After this period, 48 subjects were evaluated, with the FTP group showing a significant reduction in ALT concentrations (*P* =  0.019) compared with the placebo group. Additionally, serum aspartate aminotransferase (AST) concentrations were significantly reduced in the FTP group (*P*  = 0.02). Although γ-GT concentrations showed a tendency to decrease, alkaline phosphatase (ALP), total bilirubin (TB), and lipid concentrations remained unchanged. The study reported no severe adverse events and no abnormalities in blood glucose, total protein, albumin, BUN, or creatinine concentrations, suggesting that FTP is an effective and safe treatment for subjects with elevated ALT concentrations over a 12-wk period. Moreover, the concentrations of γ-GT indicated a tendency to be reduced, whereas the lipids and ALP were not modified. No severe abnormalities were observed in blood glucose, albumin, creatinine, total protein, and BUN concentrations [40].

### *Camellia sinensis (Theaceae)*

*Camellia sinensis* (tea) is one of the most generalized drinks among a number of populations around the world. Tea components include mainly organic acids, volatile terpenes, and polyphenols (caffeic acid, quercetin, myricetin, gallic acid, kaempferol, chlorogenic acid, and catechins), which control the taste and flavor of tea. Tea has industrial and pharmaceutical uses that include antiatherosclerotic, antihypertensive, and hypolipidemic properties [41]. The effects of fermented tea were investigated in multiple clinical trials. In an RCT, the effects of fermented tea leaf powder (by mixing loquat leaves and third crop green tea leaves) on visceral fat were investigated in healthy volunteers with obese tendency (BMI, 23–30 kg/m^2^). Following 8 wk of fermented tea ingestion at the dose of 1.05 g/d, a significant reduction in the area of visceral fat was observed, particularly in subjects <60 y of age [42]. In another RCT, the diastolic and systolic blood pressures were determined every 4 wk during 12 wk of consumption of test beverage containing 1.20 g fermented tea leaves (by mixing thinned satsuma Mandarin fruit and green tea leaves) in subjects with stage I hypertension with high-normal blood pressure. In this study, the systolic blood pressures were substantially lower in the test beverage group than in the placebo group [43].

In a single-blind study, the acute effect of fermented tea beverage (by mixing loquat leaves and third crop green tea leaves) was assessed on postprandial blood glucose concentrations in healthy volunteers. In the fermented tea beverage (3 g fermented tea with 200 mL hot water) group, elevation in postprandial blood glucose concentrations tended to be suppressed, and the suppression was substantial at 30 min in participants who exhibited a high increase of blood glucose concentrations after meals, compared with the placebo group [44]. In another RCT, the acute effect of fermented green tea on the skin temperature of the hands and feet of 60 female Korean subjects who had feelings of cold hands and feet at cold temperatures was investigated. Hand and foot skin temperatures in the fermented green tea group were significantly increased compared with the placebo group. Likewise, significantly higher temperatures (>0.8°C) were seen between the finger and the dorsum of the hand in the fermented green tea group compared with placebo [45]. In another crossover RCT, the effect of a single administration of fermented tea leaf powder (by mixing thinned satsuma mandarin fruit and tea leaves) containing 36.7 mg hesperidin on cold intolerance, shoulder stiffness, fatigue, and quality of sleep were assessed. Single feeding of tea leaf powder immediately raised the skin surface temperature of the hand and decreased the visual analog scale (VAS) score for shoulder stiffness after writing. In the long-term phase of the study, continuous feeding of the powder for 2 wk reduced the VAS scale for chronic fatigue and increased the quality of sleep, which was exhibited by decrease of the AIS (Athens Insomnia Scale) score and increased OSA-MA score (modified OSA sleep inventory MA version) [46].

### *Chamaecyparis obtusa*

**FIGURE 1:**
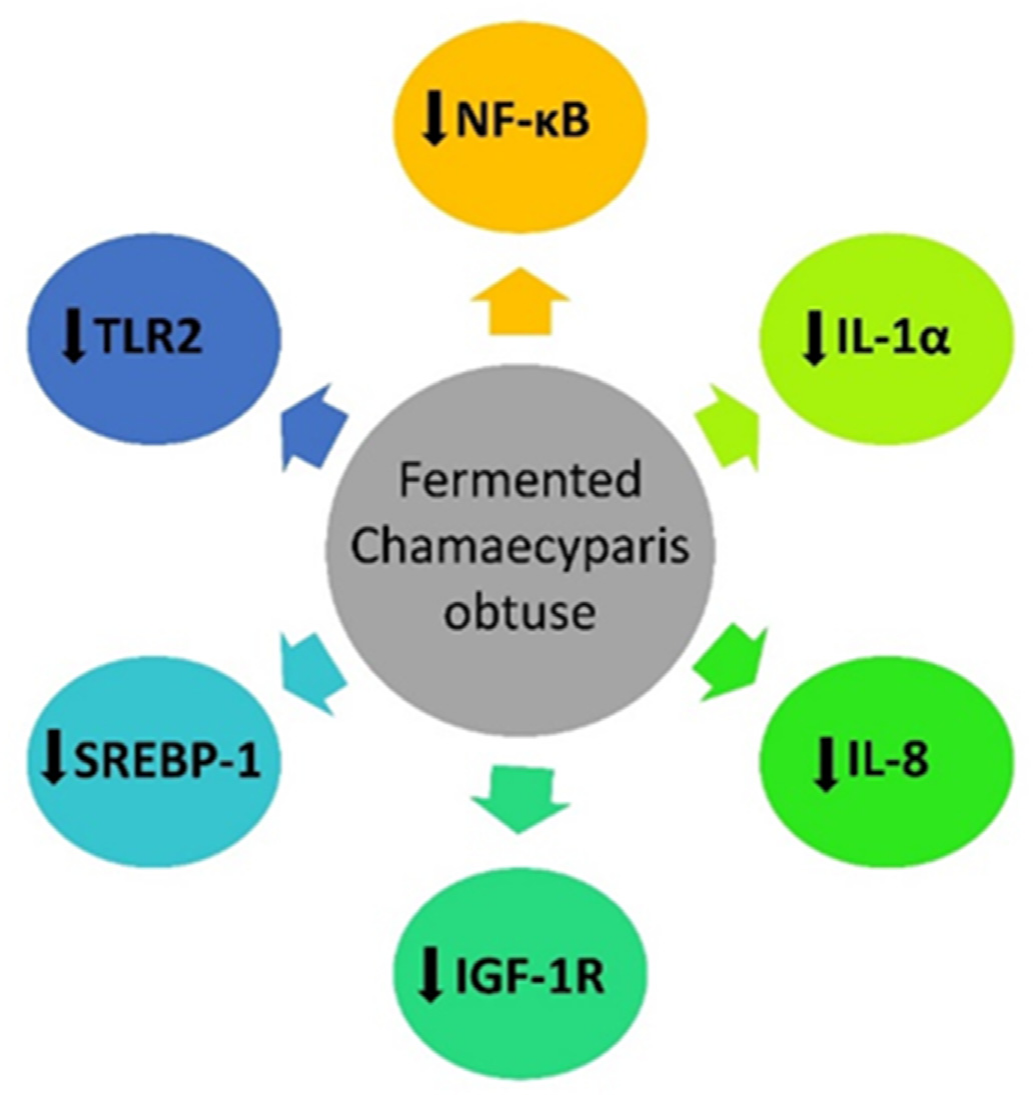
The effect of Chamaecyparis obtuse on protein expression. IGF-1R, insulin-like growth factor 1 receptor; NF-κB, nuclear factor kappa-light-chain-enhancer of activated B cells; TLR-2, toll-like receptor-2; SREBP, sterol regulatory element binding protein 1.

Extract of *Chamaecyparis obtusa*, a species of cypress found in Japan and the southern region of South Korea, has been commercially used in perfume, cosmetics, and disinfectants. Recently, its extract has been reported to have antimicrobial, antifungal, antioxidant, and anti-inflammatory effects, though its biological activities are not yet fully understood [47,48]. To compare the clinical efficacy, histopathological alterations, and safety between tea tree oil (TTO) and Lactobacillus-fermented *Chamaecyparis obtusa* (LFCO), Kwon et al. [49] conducted a split-face RCT on 32 subjects with mild-to-moderate acne. After 8 wk of treatment with TTO and LFCO creams, the inflammatory and noninflammatory lesion counts and sebum output were significantly lower on the LFCO side (one side of the face treated with LFCO) compared with the TTO side (the other side of the face treated with TTO). Compared with baseline, the overall size of the sebaceous glands decreased on the LFCO side. The protein expression of nuclear factor kappa-light-chain-enhancer of activated B cells (NF-κB) reduced earlier on the LFCO side, and IL-1α, IL-8, insulin-like growth factor 1 receptor (IGF-1R), toll-like receptor-2 (TLR-2), and sterol regulatory element binding protein-1 (SREBP-1) decreased subsequently (Figure 1). Overall, on the LFCO side, the action time was quicker than on the TTO side.

### *Triticum vulgare*

Fermented wheat germ extract (FWGE) possesses various biological activities that are utilized in medicine as adjunctive therapy in cancer. These anticancer effects are related to various significant benzoquinone derivatives that exist in FWGE, predominantly through multiple cellular molecular mechanisms. FWGE promotes immune reaction against tumor cells by reducing the expression of major histocompatibility complex class I (MHC-I) in the cell membrane and allowing cancer cells to be more efficiently identified by NK cells. Additionally, FWGE elevated the TNF-α production via macrophages, leading to improvement of immune reactions related to tumor cell angiogenesis inhibition and increased target cell apoptosis [50]. In addition, FWGE increased IL-6, IL-5, IL-2, and IL-1α expression, which have important roles in maintaining inflammatory response molecules. Moreover, FWGE can increase oxidative stress in tumor cells, stimulating the destruction of cells via free radicals, while it can affect nucleotide and carbohydrate metabolism of cancer cells. For instance, FWGE can decrease the ATP production in cells and inhibit pentose synthesis by suppressing hexokinase enzyme required for cell division. Furthermore, FWGE inhibits the activity of the ribonucleotide reductase enzyme and directly reduces nucleotide production required for the synthesis of DNA. Hence, FWGE was able to effectively reduce the proliferation of multiple types of malignant tumors and increase apoptosis of these cells [51]. In 2 open-label, controlled trials, the effect of FWGE was evaluated for cancer management. In a pilot study, Demidov et al. [52] enrolled 52 melanoma patients and randomly assigned them to receive either chemotherapy alone (control group) or FWGE (at the dose of 8.5 g/d) in addition to chemotherapy for ≤12 mo. Following an additional 7-y period, overall survival and progression-free survival were markedly elevated in the FWGE group compared with the control group. It is noteworthy that combined therapy led to lesser adverse events compared with the control group. The effects of FWGE were assessed in another trial including 55 patients with head and neck cancer. They were divided to receive either conventional oncological therapy alone or 9 g/d FWGE in addition to standard therapy. After 2 mo, FWGE treatment led to a significant reduction in circulating hydroperoxides compared with baseline and marked improvement in QOL, assessed according to Spitzer’s index, compared with the control group [53].

### *Carica papaya* L.

*Carica papaya* L. is a rich source of several types of polyphenols, including caffeic acid, quercetin, rutin, caffeoyl-hexoside, and ferulic acid. Papaya is also known as a traditional medicinal plant, and its fermented products have been found to have immune-modulatory and anti-inflammatory effects in studies in vivo and in vitro. Fermented papaya preparations (FPP) are the products of wild unripe *C. papaya* that are fermented via *Aspergillus oryzae,* and *Enterococcus faecalis*. Thus, FPPs can be categorized as synbiotic, involving probiotics and prebiotics. Many experiments have shown that live bacteria in probiotics promote human immunity through stimulation of the colon immune system [54]. A number of studies have been performed to evaluate the efficacy of FPP on various aspects of health. In one study, researchers investigated the effects of short-term consumption of FPP (6 g/d) on certain diabetes markers in a neodiabetic Mauritian population. After 2 wk of treatment, no substantial change in the concentrations of fasting blood sugar (FBS) and hemoglobin A1c (HbA1c) was perceived, though FBS slightly decreased with FPP consumption. In this trial, FPP consumption reduced risk of CVD by improving the LDL-to-HDL ratio and lowering C-reactive protein (CRP) concentrations [56]. In a crossover RCT, older adults who consumed 9 g/d of FPP for 8 wk did not show any significant effect on anthropometric parameters, metabolic outcomes, and inflammatory markers [57]. Bertuccelli et al. [55] demonstrated that 9 g/d FPP supplementation in healthy nonsmoker individuals with clinical signs of skin aging led to a substantial improvement in skin evenness, moisturization, and elasticity. Besides, SOD concentrations and nitric oxide (NO) concentration significantly increased compared with the control group. FPP substantially upregulated AQP-3 expression and downregulated cyclophilin A (CyPA) and CD147 genes compared with the control group in this randomized, double-blind, antioxidant-control group study. The effects of 7 g standardized pharmaceutical grade fermented papaya gel (intragingival pocket applications, 15 min daily) in patients with moderate-to-severe periodontitis were assessed in a 10-d RCT. At the end of 45-d follow-up, bleeding, plaque and gingival conditions, and consistent gingival pocket depth, were significantly reduced. Following fermented papaya treatment, the concentrations of proinflammatory NO metabolites (in plasma and gingival crevicular fluid), IL-1β, IL-6, and IL-10 cytokines (in blood plasma and crevicular periodontal fluid) significantly improved compared with the control group [58]. Marotta et al. [59] have shown that FPP treatment, increased lymphocyte 8-OHdG (8-hydroxydeoxyguanosine). The outcomes of this crossover RCT revealed that FPP has desirable nutraceutical effect on antioxidant defense in elderly patients, even if they did not exhibit an antioxidant-deficiency state.

### *Glycine max*

Glycine max, commonly called soybean, is a legume native to East Asia, valued for its edible bean. It consists of several types of plant proteins, soluble fibers, isoflavones, and complex carbohydrates. Soybean and soybean products such as soymilk are derived mainly from grinding and soaking the whole soybean. Other soybean products include roasted soybean, miso, boiled soybean, soy mayonnaise, soy yogurt, soy sauce, soy cheese, tofu, tamari, textured soy protein, and tempeh [60]. Fermented soybean products have garnered significant attention in the areas of nutrition and health. These products, rich in high-quality soy protein, have demonstrated their potential to positively impact human well-being. Research has shown that incorporating fermented soy products into one's diet can lead to a reduction in total cholesterol, LDL cholesterol, and triglyceride concentrations compared with animal protein consumption. Beyond their lipid-lowering effects, these soy products have exhibited promise in mitigating the effects of various health issues, including diabetes, hypertension, cardiac disorders, and certain cancer-related concerns. The increased nutritional value of fermented soy products, in contrast to their nonfermented counterparts, underscores their significance. An in-depth analysis of research findings on these products highlights their potential health benefits, which can guide future investigations to arrive at comprehensive conclusions regarding their positive impact on human health. Furthermore, a population-based prospective cohort study conducted in Japan has explored the association between fermented soy products and specific health outcomes. Among females, a notable inverse relationship was observed between the consumption of fermented soy products and risk of CVD. This association was also evident for specific items like natto and isoflavones found in fermented soy products. The study further unveiled a similar inverse link between fermented soy product intake and risk of stroke in females. However, there were no significant associations between soy products and risk of CVD in males or the overall incidence of cancer in both genders. The findings emphasize the potential health benefits, especially for females, linked to the consumption of fermented soy products such as natto. In the medical realm, fermented soybean products are increasingly recognized for their functional properties. These products, including fermented soy milk, play a vital role in regulating various health aspects, such as lipid profiles, blood vessel integrity, osteoporosis, and prostate cancer. With their ability to provide substantial soy protein concentrations and enhance human nutrition, fermented soybean products hold promise as valuable additions to a healthy lifestyle [60,61]. The effects of soybean and soymilk were investigated in several studies. In an RCT, Hwang et al. investigated the effects of fermented soybean consumption on cognitive function. In this study, 100 patients with mild cognitive impairment were assigned to consume either 800 mg/d of Lactobacillus plantarum C29-fermented soybean or placebo. At the end of a 12-wk intervention, the fermented soybean group exhibited marked improvements in the combined cognitive functions compared with the placebo group, and there were substantial associations between cognitive performance and change in serum BDNF concentration in the fermented soybean group. The lactobacilli population was markedly elevated in the fermented soybean group but not in the placebo group [62]. In another crossover trial by Tsang et al. [63], the effects of oral intake of ChemoYoung (a fermented soybean extract) from the first day of chemotherapy for ≤21 d was explored in 30 patients with advanced cancers. Compared with the control group, ChemoYoung partially (but not significantly) improved the QOL in patients and significantly restored the NK cell activity from chemotherapy toxicity. The concentrations of IL-2, 6, 10, 15, 18, T-Lymphocyte Helper/Suppressor Profile (T4/T8 ratio), and the NK cell number were not affected. In an RCT, 50 adults with mild or moderate heartburn were divided to consume ≤3 sachets (1 g) of fermented soy supplement or placebo sachets per incident heartburn (as needed) for 3 wk. After the intervention, minimal changes were detected for heartburn frequency or severity, gastro-esophageal reflux disease quality of life questionnaire (GERD-QOL), or the syndrome of gastrointestinal symptoms rating scale (GSRS). Nevertheless, in particular, QOL factors allied with discomfort caused by receiving medications, fermented soy improved concentration at work, after-meal discomfort, fear of eating, and rest compared with placebo. Also, frequency of diarrhea, bloating, and heartburn improved among washout compared with baseline for the group treated with fermented soy in comparison to placebo [64]. The effect of fermented soymilk (FSM) compared with soymilk (SM) was assessed in 2 RCTs on healthy premenopausal females. In the first study, the bioavailability of isoflavones, especially genistein, increased after 100 mL FSM consumption compared with SM consumption [65]. In the second study, intake of 100 mL FSM twice a day for 8 wk modified the gut microbiota. Both groups indicated significant improvement in skin condition. Moreover, increased concentrations of urinary isoflavones were found in both groups, and this was positively correlated with skin questionnaire scores [66].

### *Oryza sativa*

Amazake or rice koji (rice malt) is derived from glycosylated starch, from amylase action in rice malt. Amazake cake is made utilizing sake cake, which includes fermented yeast, carbohydrates, and various nutrients. Amazake provides both sake cake and rice koji. In general, rice koji contains B group vitamins (B-1, B-3, B-5, and B-12), amino acids, and glucose, as well as several bioactive compounds, products of fermentation through rice malt yeasts derived from sake cake and rice malt. Rice koji has been shown to have cholesterol-lowering activity, tyrosinase-suppressing activity, and antihypertensive properties [67]. The effects of fermented rice were assessed in several RCTs. In a clinical study, regular intake of 160 g Koji Amazake, a Japanese traditional rice fermented beverage, for 4 wk decreased systolic blood pressure [68]. In another study, intake of 300 mg/d gamma-aminobutyric acid (GABA) from fermented rice germ in 40 patients with insomnia symptoms significantly reduced sleep latency compared with the placebo group. Moreover, in the GABA treatment group, the sleep efficacy was enhanced compared with the baseline [69]. In a study by Choi et al. [70], 77 healthy participants with white blood cell counts of 4000–8000 cells/μL were randomized to receive either 3 g/d fermented rice bran (FRB) or placebo for 8 wk. At the end of the intervention, FRB treatment substantially enhanced the IFN-γ production compared to the placebo group, but FRB did not change either NK cell activity or cytokine concentrations, including IL-2, IL-4, IL-10, IL-12, and TNF-α from placebo. In another clinical study, 6–36-mo-infants with moderate-to-severe atopic dermatitis received 8 g/d fermented rice flour (FRF) or placebo (rice-powder) for 12 wk. The use of topical steroids decreased in both groups, but the reduction was significantly higher in the FRF group than in the placebo group. There were not any significant differences in cytokine concentrations, gut microbiota composition, and the SCORAD index between the groups [71].

### *Citrus* L.

Citrus-obtained flavonoids contribute many effective activities, involving anti-inflammatory, anticancer, antioxidant, antibacterial, antiviral, and supportive cardiovascular properties. Enzymatic flavonoid deglycosylation has been found to be a possible alternative signaling mechanism to enhance bioavailability of this substance and to improve the antioxidant effect of kaempferol and naringin anti-inflammatory function [71]. Citrus flavonoids have antiallergic potential. For instance, hesperidin as a flavanone glycoside is plentiful in citrus fruits and is famous for its benefits, such as suppressing mast cell degranulation, anti-inflammatory, anaphylaxis prevention, and relief of edema [72]. In 2 single-arm, nonrandomized, open-label studies conducted by Harima-Mizusawa et al. [73], the effects of 8-wk intake of 100 mL/d fermented citrus juice (FCJ) were assessed in patients with mild-to-moderate atopic dermatitis. In both studies, skindex-16 overall score and the subscores belonging to symptoms, emotions, and functioning domains significantly decreased. In subjects selected from participants of the first study, a second study showed obvious reductions in the concentrations of eosinophil cationic protein (ECP), total IgE, and specific IgEs for Japanese cedar and cypress pollen. In another RCT, volunteers with perennial allergic rhinitis were randomly divided to receive either 100 mL/d FCJ or unfermented citrus juice (control group) for 8 wk. At the end of the treatment, there were marked reductions in the total nasal symptom (TNSS) and stuffy nose score in the FCJ group compared with the control group. In addition, the FCJ group exhibited marked attenuation of Th2 cells/helper T cells, IgE, and ECP and a substantial increase of Th1 cells/Th2 cells compared with baseline [75].

### *Panax ginseng*

Many studies have suggested the anti-inflammatory effects of *Panax ginseng* and fermented red ginseng, especially against Th2-type inflammation. The effects of fermented red ginseng on allergic rhinitis were observed in such studies. In an RCT, 4-wk consumption of 1.5 g/d fermented red ginseng in 59 patients with persistent perennial allergic rhinitis led to a significant improvement in the activity and emotion of rhinitis QOL score, nasal congestion, and skin reactivity to sensitized perennial allergens compared with baseline [76]. In another clinical trial on 42 volunteers with impaired fasting glucose or type 2 diabetes, ingestion of 2.7 g/d fermented red ginseng for 4 wk markedly increased 2-h postprandial insulin concentrations and reduced postprandial glucose concentrations and the glucose area under the curve [77]. Intake of a puffed fermented Korean ginseng extract-containing beverage was studied in another RCT, where the status of stress, fatigue, and sleep were investigated in adult workers. In this trial, serum dehydroepiandrosterone sulfate (DHEA-S), serum cortisol, AIS, and a Japanese version of the Pittsburgh sleep quality index did not change. Middle-aged and older subjects aged ≥50 y were studied in a Japanese version of the profile of mood states 2nd edition-adult short (POMS2-AS) score, where fatigue-inertia from baseline to week 4 was found to be improved significantly compared with the placebo drink group [78]. Consumption of high doses of fermented ginseng powder (500 mg/d) for 12 wk in subjects with elevated ALT concentrations significantly reduced the fatigue score compared with the placebo group. Additionally, in male participants, the γ-GT and CRP concentrations decreased significantly following low-dose fermented ginseng powder (125 mg/d) compared with the placebo group. In this study, none of the treatments affected the concentrations of ALT, AST, total antioxidant capacity, and lipid profile [79].

### *Allium sativum*

Significant effects of fermented garlic extracts (FGE) on mild hepatic dysfunction in adults without underlying hepatic diseases have been confirmed in some studies. In an RCT of healthy volunteers with serum triglyceride concentrations of 120–200 mg/dL, the LDL-to-HDL ratio and serum concentration of total cholesterol and LDL substantially improved after 12 wk of daily intake of 900 mg/d fermented garlic (*P* < 0.01 compared with placebo). Anthropometric parameters (BMI, body fat, and abdominal circumference), FBS, and serum adiponectin concentration were not affected by the treatment [80]. In another clinical trial, the daily intake of 4 g/d of FGE for 12 wk in 75 adults with elevated serum γ-GT led to a significant improvement in ALT concentrations compared with the placebo group. Moreover, γ-GT tended to decrease [81].

### *Zea mays*

Fermented maize gruel is a widely available and inexpensive product, famous among people in Ghana, and is suggested as a local treatment of acute diarrhea in children. In an RCT, 108 children aged 4–27 mo with acute diarrhea and dehydration were recruited and received either oral rehydration solution (ORS) or fermented and unfermented maize solutions. The efficacy of the treatment at the end of 24 h of treatment was not significantly different, as assessed by fluid intake, stool output, stool frequency, weight gain, and duration of diarrhea. Children accepted fermented maize more readily than the unfermented solution [82].

### *Cyclopia intermedia*

It was shown that fermented *Cyclopia intermedia* used to brew honeybush tea exerts antioxidant and antiwrinkle activities by preventing the reactive oxygen species production and decreasing matrix activity. In an RCT, the effects of low and high doses of fermented honeybush extract intake were investigated against skin aging in 115 subjects with crow’s feet wrinkles. Following 12 wk intervention, both the low-dose (400 mg/d) and high-dose (800 mg/d) groups exhibited substantial improvement in transepidermal water loss (TEWL), global skin wrinkle grade, as well as the skin hydration and elasticity compared with the placebo group, but no substantial differences were observed between low- and high-dose groups [83].

### *Vitaceae* spp.

Grape seeds are the part of the fruit with the highest concentration of bioactive molecules. Multiple studies found that between the various part of grape fruit, seeds represent the maximum antioxidant function, accompanied by the skin and pulp. Grape seeds have suitable biological potential that might be exploited by extraction of bioactive substances with greater additional value than utilizing biomass for energy, with the aim to acquire semifinished products and extracts appropriate for agronomic, feed, nutraceutical, cosmetics, food, and pharmaceutical goals [84]. Hardaliye, an ancient and relatively obscure traditional beverage, is crafted from the remnants of red grapes used in winemaking. This beverage's creation involves lactic acid fermentation, with the incorporation of sour cherry leaves and mustard seeds, available in different concentrations and forms, whether heat-treated, ground, or whole. Hardaliye boasts a brief shelf life, prompting recent endeavors to employ innovative processing methods to extend its longevity. In an RCT, 89 healthy adults were randomly assigned into 3 groups: high hardaliye (HH, 500 mL/d), low hardaliye (LH, 250 mL/d), and a control group. The antioxidant status was evaluated at baseline as well as after 40 d of supplementation. At the end of the study, both HH and LH groups revealed significant reduction in diene conjugate and MDA concentrations compared with the control group. Moreover, a dose-response reduction in homocysteine concentration was observed. Slight elevations in the concentrations of total antioxidant capacity and vitamin C were found [85].

### *Vaccinium myrtillus* L.

Bilberry (*Vaccinium myrtillus* L.) is rich in anthocyanins, amounting to ∼2000–3500 mg/kg fresh weight. The most frequent monomeric anthocyanins found in bilberry include galactosides, cyanidin, glucosides, and arabinosides of delphinidin, peonidin, petunidin, and malvidin. Fermented bilberry products are increasingly valued for their potential health benefits [86]. In an RCT, following 3-mo fermented bilberries (∼10 g fresh bilberries/d) consumption in patients with hypertension, neither the diversity nor the content of oral and fecal microbiota was significantly changed [87]. In a crossover study, the effects of fermented bilberry extract on visual outcomes in healthy volunteers with myopia were assessed. Bilberry ingestion at the dose of 400 mg/d for 4 wk led to a significant elevation in the mesopic area under the log contrast sensitivity function (AULCSF) and the mean amplitude of accommodation compared with baseline. Nonetheless, other indices such as visual acuity, pupil constriction rate, refraction, and mesopic contrast sensitivity were not affected [88].

## Conclusion and Perspectives

**FIGURE 2:**
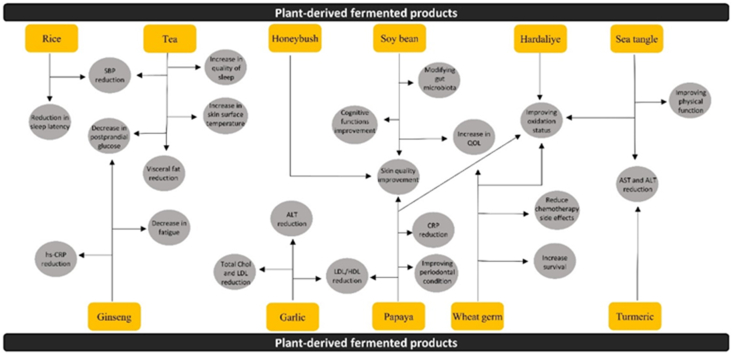
A summary of the effects of different types of fermented plants products. ALT, alanine transaminase; AST, aspartate aminotransferase; Chol, cholesterol; CRP, C-reactive protein; hs-CRP, high-sensitivity C-reactive protein; QOL, quality of life

The multifaceted nature of plant-derived fermented products involves a constellation of factors that intricately shape their health-related outcomes. To address the crucial questions regarding the influence of various factors on the health benefits of these products, available data from diverse sources have been reviewed, including publications in PubMed and Scopus. First and foremost, the type of bacteria employed for fermentation plays a pivotal role in dictating the nutritional and bioactive composition of the final product. Different bacterial strains bring about distinct metabolic pathways, leading to variations in the synthesis of essential compounds. LAB, for instance, has been widely recognized for its involvement in fermenting plant-based materials and its ability to generate bioactive molecules such as lactic acid and specific peptides with potential health benefits. The choice of microbial cultures, therefore, can significantly affect the health-promoting properties of the fermented product. Additionally, the source of the plant materials is a critical determinant. Variations in the botanical origin, species, and geographical location can impart differences in the nutrient profiles and phytochemical content of the raw materials. These disparities, in turn, can influence the types and concentrations of bioactive compounds synthesized during the fermentation process. Whether it is seaweed from the coasts of Korea or soybeans from the heartland of Japan, the origin of the plant material is a fundamental consideration. Moreover, the fermentation environment itself, encompassing factors like temperature, pH, and duration, plays an indispensable role in shaping the final product. These conditions can modulate the growth and activity of microorganisms, affecting the production of specific metabolites that underpin the health benefits. Optimal fermentation conditions are essential for achieving the desired functional attributes in the end product. In light of the intricate interplay of these factors, it becomes evident that a holistic understanding of the entire fermentation process, from the selection of microbial strains to the sourcing of raw materials and the control of environmental conditions, is indispensable in unraveling the complex relationship between plant-derived fermented products and their health-promoting potential. In this report, we reviewed the potential health benefits of plant-derived fermented products such as garlic, bilberry, turmeric, ginseng, and others on various conditions, such as skin diseases, atherosclerosis, CVD, and others (Figure 2). Plant-derived fermented products have been used as foods or beverages in humans' lifestyles since ancient times. In most cases, plant-derived fermented products were considered to be superior to the unprocessed plant compounds for enhanced efficacy. Although studies on plant-fermented products demonstrated their promising abilities to improve various disease conditions clinically (Table 1) [17,18,38,43,45,49,52,53,[55], [56], [57], [58], [59],[62], [63], [64], [65], [66],[68], [69], [70], [71],[74], [75], [76], [77], [78], [79], [80], [81], [82], [83],[85], [86], [87], [88], [89], [90], [91], [92], [38], [39]], it is important to define the differences between pure extracts or fermented products in more controlled clinical studies. Despite the demonstrated efficacy of many fermented products, the exact mechanisms of action remain elusive and require further investigation. Additional studies are needed to elucidate the signaling pathways involved and to uncover the specific mechanisms underlying their therapeutic effects. Fermentation products generally modulate inflammatory signaling pathways and immunomodulatory biomarkers such as preinflammatory and proinflammatory cytokines. For instance, it was shown clinically that fermented ginseng powder significantly reduced the hs-CRP concentrations [76], whereas rice bran exo-biopolymer enhanced IFN-γ concentrations [70]. Elucidation of the precise signaling pathways would provide appropriate alternative and complementary approaches to improving health conditions while attenuating clinical symptoms of various disorders. Despite the significant amount of evidence suggesting that fermented products improve a number of disease symptoms, additional studies are warranted to investigate any long-term adverse effects of each plant-derived fermented product in such health conditions.

**TABLE 1:** Clinical studies on plant-derived fermented products

Study design	Intervention (case/control)	Duration	Population	Number of patients (case/control)	Assessments	Outcome	Reference
Randomized, controlled	1. High hardaliye (HH) (500 mL/d, by mouth) 2. Low hardaliye (LH) (250 mL/d, by mouth) 3. Control group (none)	40 d	Healthy adults	HH: 45 LH: 35 Control: 20	DC, MDA, vitamin C, TAC, homocysteine concentrations	HH and LH groups revealed significantly reduced DC, homocysteine, and MDA concentrations.	[85]
Randomized, double-blind, placebo-controlled	1. Fermented sea tangle (FST) (6 capsules each contain 250 mg FST/d) 2. Placebo (6 capsules/d)	4 wk	Healthy male volunteers with high concentrations of γ-GT	24/group	Serum γ-GT, MDA, CAT, SOD, and GPx activities	FST markedly decreased serum concentrations of γ-GT and MDA and significantly augmented SOD and CAT activities	[17]
Randomized, double-blind, placebo-controlled	1. FST (6 capsules each contain 250 mg FST/d) 2. Placebo (6 capsules/d)	4 wk	Healthy male volunteers with high concentrations of γ-GT	24/group	γ-GT, AST, ALT, ALP, LDH, BUN, creatinine, 8-isoprostane, 8-OHdG, and protein carbonyl contents	FST significantly decreased γ-GT, AST, ALT, LDH, BUN, 8-isoprostane, 8-OHdG, and protein carbonyl content	[18]
Randomized, double-blind, controlled split-face	1. 5% cream of LFCO (Topical, twice daily, one side) 2. 5% cream of TTO (Topical, twice daily, one side)	8 wk	Participants (male and female) with mild-to-moderate acne	32	Acne counts, sebum secretion, size of sebaceous glands, IL-1α, IL-8, IGF-1R, TLR-2, and SREBP-1	1. Inflammatory and noninflammatory lesion counts and sebum output were significantly lower on LFCO. 2. The overall size of the sebaceous glands decreased on the LFCO 3. Protein expressions of NF-κB reduced earlier on the LFCO side, and IL-1α, IL-8, IGF-1R, TLR-2, and SREBP-1 decreased subsequently.	[49]
Randomized, placebo-controlled	1. Fermented green tea (by mouth, 100 mL hot water + a tea bag) 2. Placebo (hot water) (by mouth, 100 mL)	1 h	Female Korean subjects with cold hypersensitivity	30/group	Cold hypersensitivity	1. Fermented green tea remarkably increases skin temperature of the hands and feet more than control group 2. The temperature difference between the finger and the dorsum of the hand was significantly lower in the fermented green tea group	[45]
Randomized, double-blind	1. Fermented papaya preparation (FPP-treated group) (4.5 g/d sachet, sublingually, twice daily) 2. An antioxidant cocktail (4.5 g/d sachet, sublingually, twice daily)	90 d	Healthy nonsmoker individuals with clinical signs of skin aging	30/group	Skin biometric parameters, NO, MDA, SOD, and gene expression of AQP-3, CyPA, CD147, and progerin	1. Significant improvement in skin evenness, moisturization, and elasticity 2. MDA decreased, and SOD increased significantly in FPP group 3. NO concentration was significantly higher in FPP group 4. FPP group showed significant upregulation of AQP-3 and downregulation of CyPA and CD147 genes	[57]
Randomized, double-blinded, placebo-controlled	1. Low dose of fermented honeybush extract (400 mg/d, by mouth, twice daily) 2. High dose of fermented honeybush extract (400 mg/d, by mouth, twice daily) 3. Placebo (dextran, by mouth, twice daily)	12 w	Subjects with moderate-to-severe crow’s feet wrinkles	40/group	Skin wrinkle grade, elasticity, hydration, and TEWL	Skin wrinkle grade and skin hydration and elasticity were significantly improved, and TEWL substantially decreased in both low-dose and high-dose groups	[83]
Prospective, randomized, double-blind, 2-arm placebo-controlled	1. Fermented rice flour (FRF) (8 g/d, by mouth) + topical steroids 2. Placebo (rice-powder) (8 g/d, by mouth) + topical steroids	12 w	Infants with moderate-to-severe atopic dermatitis, aged 6–36 mo	Treatment group: 27 placebo: 26	Gut microbiota composition Serum cytokines Steroid-usage	The use of topical steroids decreased significantly more in FRF group, and Gut microbiota composition had no difference	[71]
Two-Single-arm, nonrandomized, open-label	Fermented citrus juice (by mouth, 100 mL)	8 wk	Subjects with mild-to-moderate atopic dermatitis	52	ECP, IgE	Citrus fermented juice containing heat-killed LP0132 improves QOL in patients via attenuation of IgE and ECP	[74]
Prospective, randomized, double-blind, and placebo-controlled	1. GABA from fermented rice germ (RFE-GABA) (300 mg/d, by mouth) 2. Placebo (Maltodextrin)	4 wk	Patients who complained of insomnia symptoms	Treatment group: 30 placebo: 10	Subjective and objective improvements in sleep quality	Improved subjective sleep quality and objective sleep efficacy without severe adverse events	[69]
Randomized, double-blind, and placebo-controlled	1. Doenjang (Korean fermented soy bean paste) (9.8 g/d, by mouth, 3 times daily) 2. Placebo (a placebo pill/d, by mouth)	12 wk	Overweight subjects with the PPAR-γ C1431T Polymorphism	Treatment group: 26 placebo: 25	Antiobesity and antioxidant effects	Decreased visceral fat accumulation and aging significantly in subjects with PPAR-γ polymorphism	[89]
1. Randomized, double-blind, placebo-controlled, parallel-group	1. Rice bran exo-biopolymer (RBEP) (6 capsules each contain 500 mg RBEP/d) 2. Placebo (6 capsules each contain 500 mg placebo/d)	8 wk	Healthy participants with white blood cell counts of 4000–8000 cells/μL	40/group	NK cell activity, IFN-γ, IL-2, 4,10,and 12, TNF-α	1. RBEP supplementation significantly increased IFN-γ production 2. RBEP did not affect either NK cell activity or cytokine concentrations, including IL-2, IL-4, IL-10, IL-12, and TNF-α	[70]
Randomized, double-blind, placebo-controlled	1. Fermented soy [≤3 sachets (1 g)/heartburn incident, by mouth] 2. Placebo (maltodextrin) [≤3 sachets (1 g)/heartburn incident, by mouth]	3 wk	Adults with mild or moderate heartburn with ≥2 d/wk used OTC products for heartburn	Treatment group: 23 placebo: 27	Heartburn severity or frequency, GSRS, GERD-QOL	1. No significant differences in heartburn severity or frequency, GSRS syndromes, or GERD-QOL. 2. Frequency of heartburn, diarrhea, and bloating improved	[64]
Randomized, double-blind, placebo-controlled, parallel-group	1. Garlic fermented with Monascus pilosus (MGFE) (4 capsules each contain 225 mg/d) 2. Placebo (4 capsules, each containing 300 mg/d with an essence color similar to that of MGFE)	12 wk	Healthy individuals with serum triglyceride concentrations of 120–200 mg/dL	Treatment group: 28 placebo: 27	Serum lipid contents	Decreased triglyceride and total cholesterol in serum, and the LDL-to-HDL ratio substantially improved	[80]
Randomized, double-blind, placebo-controlled	1. Lactobacillus plantarum C29-fermented soybean (DW2009) (800 mg/d, by mouth) 2. Placebo (800 mg/d, by mouth)	12 wk	Individuals with mild cognitive impairment	40/group	Cognitive function, serum BDNF concentrations	Enhance cognitive function through increased serum BDNF concentrations after consumption of DW2009	[62]
Randomized crossover	1. Unfermented whole grain rye crisp bread (URCB) 2. Fermented whole grain rye crisp bread (RCB) 3. Refined wheat crisp bread (WCB)	4 h	Healthy volunteers with BMIof 18–31.4 kg/m^2^	23	Appetite and postprandial insulin response	URCB induced lower postprandial insulin response between 0–230 min compared with RCB and WCB, respectively	[90]
Randomized, double-blind, placebo-controlled	1. Fermented red ginseng (FRG) (3 capsules daily (250 mg/capsule, twice daily)) 2. Placebo (starch) (3 capsules daily (250 mg/capsule, twice daily))	4 wk	Patients with persistent perennial allergic rhinitis	59	TNSS duration score RQoL score Skin reactivity to inhalant allergens	FRG reduced skin reactivity to sensitized perennial allergens and improved RQoL and nasal congestion	[76]
Randomized, double-blind, placebo-controlled	1. Fermented Saccharina japonica (FSJ) extract (2 capsules daily 500 mg/capsule) 2. Placebo (cellulose, lactose, and magnesium stearate) (2 capsules daily 500 mg/capsule)	4 wk	Healthy participants	Treatment group: 33 placebo: 36	Cognitive function SOD	Improve cognitive function via regulation SOD system	[91]
Randomized, double-blind, placebo-controlled	1. Low dose of fermented ginseng powder (GBCK25) (125 mg/d) 2. High dose of fermented honeybush extract (500 mg/d) 3. Placebo	12 wk	Participants with elevated ALT concentrations	30/group	Liver functions via ALT, γ-GT, AST, hs-CRP, multidimensional fatigue	1. hs-CRP concentrations decreased significantly following low-dose GBCK25 compared with placebo group 2. High-dose GBCK25 significantly reduced the fatigue score compared with the placebo group	[79]
Randomized, placebo-controlled, crossover	1. Fermented bilberry extract (400 mg/d) 2. Placebo (rapeseed oil) (400 mg/d)	4 wk	Healthy volunteers with myopia	30	Visual acuity, refraction, pupil constriction rate, accommodation, and CS, AULCSF	The mesopic AULCSF and the accommodation increased significantly	[88]
Open randomized	1. Standardized fermented papaya gel (SFPG) (7 g/d, intragingival pocket) 2. Control	10 d	Patients with moderate-to-severe periodontitis	Treatment group: 39 placebo: 45	Plaque and gingival conditions, bleeding, gingival pocket depth, GCF and plasma concentrations of NO metabolites, IL-1β, 6, 10	1. Bleeding, plaque and gingival conditions, and consistent gingival pocket depth were significantly reduced. 2. The concentrations of NO metabolites and IL-1β, IL-6 reduced, whereas IL-10 increased	[58]
Randomized, double-blind, placebo-controlled	1. Japanese traditional rice fermented beverage (160 g/d) 2. Placebo (160 g/d)	4 wk	Patients with high blood pressure	10/group	Systolic and diastolic blood pressure	Systolic blood pressure significantly decreases	[68]
Randomized, double-blind, placebo-controlled	1. Fermented garlic extract (40 g/d, by mouth) 2. Placebo (160 g/d, by mouth)	12 wk	Adult patients with elevated serum γ-GT	Treatment group: 36 placebo: 39	γ-GT, AST, ALT, fatigue scale score	Improvement in concentrations of γ-GT and ALT	[81]
Randomized, double-blind, placebo-controlled	1. Fermented turmeric powder (FTP) (6 capsules daily 500 mg/capsule, 3 times daily) 2. Placebo (6 capsules daily 500 mg/capsule, 3 times daily)	12 wk	Subjects with mild-to-moderate elevated ALT concentrations (40 IU/L–200 IU/L)	Treatment group: 26 placebo: 22	γ-GT, AST, ALT, ALP, lipid profile, glucose, total bilirubin, BUN, creatinine	1. Significant reduction in ALT and AST concentrations in FTP group 2. γ-GT demonstrated tendency to decrease 3. No abnormality in lipid, total bilirubin, creatinine, and BUN	[40]
Randomized, double-blind, placebo-controlled	1. Puffed fermented Korean ginseng extract-containing drink (FGD) (containing 10 mg of ginsenosides/d) 2. Placebo (same volume of drink/d)	8 wk	Adult workers	38	Fatigue, stress, sleep	POMS2-AS scores show markedly improved mental fatigue	[78]
Randomized, placebo-controlled, crossover	1. FPP (9 g/d, by mouth) 2. Placebo (granulated sugar, 9 g/d, by mouth)	8 wk	Old healthy person (70–100 y)	29	Anthropometric parameters, glucose, CRP, MPO, IL-6 AST,ALT, ALP, lipid profile, total bilirubin, BUN, creatinine	1. No significant effect on antithrombotic and metabolic results. 2. No adverse impact on biomarkers	[56]
Randomized, placebo-controlled, crossover	1. FPP (9 g/d, by mouth) 2. Placebo (granulated sugar, 9 g/d, by mouth)	3 mo	Elderly patients without major diseases	54	GSTM1 and redox status	Improving antioxidant defense in elderly patients, even without any overt antioxidant-deficiency state	[59]
Randomized, double-blind, placebo-controlled	1. Fermented soymilk (FSM) (100 mL/d, by mouth, twice daily) 2. Soymilk (SM) (100 mL/d, by mouth, twice daily)	12 wk	Healthy premenopausal females	FSM: 27 SM: 25	Fecal microbiota, urinary isoflavone concentrations, skin condition	FSM improves skin condition via increased concentrations of isoflavone absorption in the body	[66]
Randomized, double-blind, placebo-controlled, single-dose, crossover	1. FSM (100 mL/d, by mouth) 2. SM (100 mL/d, by mouth)	5 h	Healthy premenopausal females	FSM: 4 SM: 3	Serum isoflavones	Total isoflavones were significantly higher for ≤5 h after the intake of FSM compared with SM	[65]
Randomized, double-blind, placebo-controlled	1. Fermented tea leaves (1.2 g/d, by mouth) 2. Placebo (1.2 g/d, by mouth)	12 wk	Subjects with high-normal blood pressure and stage I hypertension	39	Systolic and diastolic blood pressure	Systolic blood pressure reduced significantly	[43]
Randomized, double-blind, placebo-controlled	1. Fermented cow milk (7 g/d in 150 mL, by mouth) 2. Fermented rice (7 g/d in 150 mL, by mouth) 3. Placebo (maltodextrins, 7 g/d in 150 mL, by mouth)	3 mo	Healthy children (aged 12–48 mo)	1. Fermented cow milk: 137 2. Fermented rice: 118 3. Placebo: 122	α- and β-defensins and cathelicidin LL-37, and secretory IgA, appearance of CIDs	1. Presenting acute gastroenteritis and upper respiratory tract infections were lower in Fermented cow milk and Fermented rice groups 2. Prevents CIDs in children via stimulation of innate and acquired immunity	[92]
Randomized, double-blind, placebo-controlled	1. FRG (3 capsules daily 900 mg/capsule, 3 times daily) 2. Placebo (3 capsules daily 900 mg/capsule, 3 times daily)	4 wk	Subjects with impaired fasting glucose or type 2 diabetes	21/group	Fasting and postprandial glucose, insulin, and postprandial insulin	1. Significant reduction in postprandial glucose concentrations 2. Increase in postprandial insulin concentrations	[77]
Randomized, controlled	1. FPP (6 g/d, by mouth) 2. Placebo (6 g/d, by mouth)	14 wk	Neodiabetic subjects	101	LDL-to-HDL ratio, CRP, uric acid, antioxidant status	1. The LDL-to-HDL ratio and the concentrations of CRP and uric acid significantly improved 2. Improve the general health status of several organs targeted by oxidative stress	[55]
Open-label, controlled	1. Conventional oncological therapy + fermented wheat germ (FWGE) (9 g/d, by mouth) 2. Conventional oncological therapy alone	2 mo	Patients affected by head and neck tumors (stage IIIa, IIIb, IV)	Treatment group: 26 placebo: 29	Spitzer index, Oxidative stress	Concentrations of Oxidative stress significantly decreased, and QOL improved	[53]
Randomized, open-label, self-controlled, crossover	1. Fermented soybean extract + chemotherapy (8 mL/d, by mouth) 2. Chemotherapy	3 wk	Patients with advanced cancers	30	NK cell activity, T4/T8 ratio, NK cell, QOL, IL-2, IL-6, IL-10, IL-15, IL-18	Fermented soybean extract partially improved QOL and significantly restored the NK cell activity from chemotherapy toxicity.	[63]
Randomized, double-blind, placebo-controlled	1. Fermented bilberries (10 g/d, by mouth) 2. Live *Lactobacillus plantarum* strain DSM 15313 (10 g/d, by mouth) 3. Placebo (10 g/d, by mouth)	3 mo	Hypertensive patients	47–48/group	Blood pressure, oral, and fecal microbiota	No significant change	[87]
Randomized, controlled	1. ORS (752 mL, by mouth) 2. Fermented maize solution (656 mL, by mouth) 3. Unfermented maize solution (348.9 mL, by mouth)	24 h	Children (aged 4–27 mo with acute diarrhea and mild to moderately severe dehydration)	35/group	Serum osmolality and electrolyte concentrations, stool output, stool frequency, weight gain, and duration of diarrhea	No significant differences in the efficacy of the various treatments	[82]
Randomized, pilot, phase II	1. FWGE (8.5 g/d, by mouth) 2. Control (DTIC-based adjuvant chemotherapy)	1 y	Stage III skin melanoma patients receiving adjuvant chemotherapy	26/group	Overall survival, progression-free survival, adverse events	Overall survival and progression-free survival were significantly higher FWGE and fewer adverse reactions	[52]

Abbreviations: DC, dien conjugate; MDA, malondialdehyde; TAC, total antioxidant capacity; LFCO, Lactobacillus-fermented *Chamaecyparis obtuse*; TTO, tea tree oil; TEWL, transepidermal water loss; ECP, eosinophil cationic protein; GABA, gamma-aminobutyric acid; GSRS, Gastrointestinal Symptoms Rating Scale; GERD-QOL, gastro-esophageal reflux disease quality of life Questionnaire; OTC, over-the-counter; BDNF, brain-derived neurotrophic factor; RQoL, Rhinitis Quality of Life; TNSS, total nasal symptom; AULCSF, area under the log contrast sensitivity function; CS, mesopic contrast sensitivity; GCF, gingival crevicular fluid; NO, nitric oxide; GSTM1, glutathione-S transferase M1; CID, common infectious disease; FRG, fermented red ginseng; FPP, fermented papaya preparation; QOL, quality of life; ORS, oral rehydration solution; FWGE, fermented wheat germ extract; DTIC, dacarbazine; NK cells, natural killer cells; T4/T8 ratio, T-lymphocyte helper/suppressor profile; CRP, C-reactive protein; FSM, fermented soymilk; SM, soymilk; ALT, alanine transaminase; AST, aspartate aminotransferase; MPO, myeloperoxidase; ALP, alkaline phosphatase; BUN, blood urea nitrogen; FGD, puffed fermented Korean ginseng extract-containing drink; POMS2-AS, Japanese version of the profile of mood states 2nd edition-adult short; FTP, fermented turmeric powder; γ-GT, gamma-glutamyl transferase; IU, International Unit; SFPG, standardized fermented papaya gel; fermented ginseng powder: GBCK25; hs-CRP, high-sensitivity C-reactive protein; FSJ, fermented *Saccharina japonica*; SOD, superoxide dismutase; TNSS, total nasal symptoms score; URCB, unfermented whole grain rye crisp bread; RCB, fermented whole grain rye crisp bread; WCB, refined wheat crisp bread; DW2009, *Lactobacillus plantarum* C29-fermented soybean; MGFE, garlic fermented with *Monascus pilosus*; RBEP, Rice bran exo-biopolyme; IFN-γ, interferon gamma; PPAR-γ, peroxisome proliferator- activated receptor gamma; FRF, fermented rice flour; AQP-3,aquaporin 3; CypA, cyclophilin A; IGF-1R, insulin-like growth factor 1 receptor; TLR, toll-like receptor; SREBP-1, sterol regulatory element binding protein-1; NF-κB, nuclear factor kappa-light-chain-enhancer of activated B cells; 8-OHdG, 8-hydroxy-2'-deoxyguanosine; FST, fermented sea tangle; CAT, catalase; GPx, Glutathione peroxidase.

## Understanding the Nuances in Assessing Health Benefits of Plant-Derived Fermented Products

In our extensive exploration of the potential health benefits associated with plant-derived fermented products, we recognize the importance of evaluating both strengths and limitations present in the studies reviewed. These studies collectively offer a diverse panorama of potential health impacts that span across various dietary preferences and cultural contexts. The inclusion of several clinical trials enhances the credibility of reported findings. Furthermore, studies revealing biologically plausible mechanisms underscore the potential of these fermented products as therapeutic agents. However, although potential mechanisms have been identified, further research is needed to fully understand and validate their role in mediating therapeutic outcomes. However, amidst these strengths, our review has illuminated certain limitations prevalent in existing literature. Heterogeneity in study designs, encompassing differences in sample sizes, study durations, and outcome measures, presents challenges in making direct comparisons and drawing overarching conclusions. The predominantly short-term nature of these studies restricts our comprehension of the long-term effects and safety profiles of these products. Therefore, a crucial need exists for more extensive investigations to delineate potential adverse effects and ascertain the sustainability of these health benefits over extended periods. Moreover, the variability in product composition, influenced by factors like bacterial strains and source materials, contributes to discrepancies in findings and impedes the formulation of universally applicable conclusions. The disparity in effect sizes among studies, ranging from statistically significant but small improvements to more substantial effects, emphasizes the necessity of considering both statistical and clinical significance. Taking into account these factors, our review emphasizes the importance of adopting a thoughtful approach to interpreting the findings of the studies discussed. It is crucial to acknowledge both the strengths and limitations inherent in each study, as well as to discern the clinical significance of effect sizes in the context of enhancing health outcomes. By embracing these nuances, we can develop a more thorough understanding of the potential health advantages offered by plant-derived fermented products. This nuanced perspective will not only contribute to a more comprehensive comprehension of these products but also lay the groundwork for future research initiatives aimed at uncovering their complete therapeutic potential.

## Author contributions

The authors’ responsibilities were as follows –; AHA, BDR, AS: conceptualization; DK, KN: writing-original draft; SM, BDR, MA, TJ, AHA, AS: writing-review and editing; and all authors read and approved the final manuscript.

## Conflicts of interest

The authors report no conflicts of interest.

## Funding

The authors reported no funding received for this study.
